# Disruption of macrophage pro-inflammatory cytokine release in Crohn's disease is associated with reduced optineurin expression in a subset of patients

**DOI:** 10.1111/imm.12338

**Published:** 2014-12-08

**Authors:** Andrew M Smith, Gavin W Sewell, Adam P Levine, Thean S Chew, Jenny Dunne, Nuala R O'Shea, Philip J Smith, Penelope J Harrison, Carol M Macdonald, Stuart L Bloom, Anthony W Segal

**Affiliations:** 1 Microbial Diseases, Eastman Dental Institute, University College LondonLondon, UK; 2 Division of Medicine, University College LondonLondon, UK; 3 Department of Gastroenterology, UCLH NHS Foundation TrustLondon, UK

**Keywords:** cytokine, gene expression, inflammatory bowel disease, immunodeficiency, inflammation

## Abstract

Crohn's disease (CD) is a complex and highly heterogeneous chronic inflammatory disorder, primarily affecting the gastrointestinal tract. Genetic and functional studies have highlighted a key role for innate immunity in its pathogenesis. Profound systemic defects in innate immunity and acute inflammation are understood to result in markedly delayed clearance of bacteria from the tissues, leading to local chronic granulomatous inflammation and compensatory adaptive immunological changes. Macrophages, key orchestrators of acute inflammation, are likely to play an important role in the initial impaired innate immune response. Monocyte-derived macrophages from CD patients stimulated with *Escherichia coli* were shown to release attenuated levels of tumour necrosis factor and interferon-*γ* with normal secretion of interleukin-8 (IL-8), IL-10 and IL-6. In controls, the secretion of these cytokines was strongly positively correlated, which was not seen with CD macrophages. The transcriptomes of CD and control macrophages were examined in an attempt to understand the molecular basis of this defect. There were no differentially expressed genes identified between the two groups, consistent with genetic heterogeneity; however, a number of molecules were found to be under-expressed in subgroups of CD patients. The most common of these was optineurin (OPTN) which was under-expressed in approximately 10% of the CD patients. Reduced OPTN expression coincided with lower intracellular protein levels and diminished cytokine secretion after bacterial stimulation both in the patients and with small interfering RNA knockdown in THP-1 cells. Identifying and studying subgroups of patients with shared defective gene expression could aid our understanding of the mechanisms underlying highly heterogeneous diseases such as CD.

## Introduction

Crohn's disease (CD) is a chronic inflammatory disorder, primarily affecting the gastrointestinal tract. It arises through an aberrant interaction between the bowel contents and the immune system, although the mechanistic basis of disease pathogenesis remains poorly understood.^1^ Family and twin studies demonstrate that the disease has a strong genetic component; the disease concordance is nearly 30% in monozygotic twins compared with 2% in dizygotic twins.^2^ Characterization of the genetic basis of CD has gathered pace in recent years through the use of genome-wide association studies^3^ and subsequent meta-analyses which, to date, have identified 140 loci associated with CD.^4^ It has been estimated that these susceptibility variants account for approximately 14% of the total disease variance.^4^ Although many of the associated loci are suspected of influencing the immune system, the functional variants within each locus and the underlying pathogenic mechanisms remain unclear.^5^ The most significantly enriched Gene Ontology category associated with the 140 genome-wide association study loci was the regulation of cytokine production, specifically tumour-necrosis factor-*α* (TNF-*α*), interferon-*γ* (IFN-*γ*), interleukin-12 (IL-12) and IL-10 signalling.^4^

It is becoming increasingly evident that most cases of CD result from an innate immunodeficiency^6^ and a primary pathogenic defect has been shown in the monocyte-derived macrophage (MDM) response to bacteria.^7^–^9^ Macrophages orchestrate cellular responses against a complex and diverse range of intestinal microbial insults via pattern recognition receptors such as Toll-like receptors (TLR) and NOD-like receptors (NLR).^10^,^11^ Activation through these receptors leads to the secretion of inflammatory mediators that recruit leucocytes from the surrounding microcirculation. It has been postulated that the dysregulated macrophage response to bacteria may be central to the pathogenesis of CD^12^ and a number of studies on monocytes/macrophages from CD patients have reported reduced pro-inflammatory cytokine release, in response to NLR, TLR and bacterial stimulation.^7^–^9^,^13^ CD-associated polymorphisms in NOD2 (L1007finsC, R702W, G908R) result in impaired immunity, through defective nuclear factor-*κ*B activation and deficient secretion of pro-inflammatory cytokines in response to muramyl peptide (a component of bacterial peptidoglycan).^13^,^14^ However, the majority of CD patients do not carry these polymorphisms in NOD2 and respond normally to muramyl peptide.^9^,^13^ Attenuated secretion of pro-inflammatory cytokines by macrophages is likely to have a profound effect on the innate immune response in these individuals. The previously reported impaired neutrophil recruitment and delayed bacterial clearance reported in patients with CD may well be secondary to abnormal cytokine secretion.^9^,^15^,^16^ The retention of the undigested bacteria at sites of ingress was proposed to be the driving force for the ensuing chronic granulomatous inflammation, and secondary adaptive immune response characteristic of CD.^12^

Cytokine secretion is a highly complex and tightly regulated process.^17^ Different cell types have been shown to possess unique activation and secretory pathways, and furthermore, multiple exocytosis pathways have been identified for individual cytokines.^18^ To understand the defective macrophage secretory mechanisms associated with CD we have concentrated our study on TNF-*α*, IFN-*γ*, IL-6, IL-8 and IL-10, which have been shown to be abnormal in CD^9^,^15^,^19^ and used transcriptomic analyses in an attempt to identify the responsible molecules.

## Materials and methods

### 

#### Ethics approval and patient recruitment

Adult patients with a definitive diagnosis of CD confirmed using standard diagnostic criteria were recruited from the Gastroenterology outpatient clinic at University College London Hospitals NHS Foundation Trust (UCLH). All patients had quiescent disease (CD; Harvey–Bradshaw Activity index ≤ 3).^20^ The demographics of patients included in this study are outlined in Table 1.

**Table 1:** Patient demographics

	HC	CD	HC versus CD *P*-value	CD^low^	CD versus CD^low^ *P*-value
*n*=	42	58		7	
Gender (M:F)	22 : 20	29 : 29	0·84	6 : 1	0·11
Mean age ± SD	37·8 ± 10·4	39·1 ± 14·0	0·64	49·3 ± 16·8	0·06
Age range	22–61	19–69		24–66	
Current smokers	4	5	1·00	1	0·51
Treatment					
No medication		17		2	1·00
5-Aminosalicylates		36		3	0·20
Immunosuppressants		8		2	0·59
Biological agents		2		0	1·00
Disease location					
L1		15		1	0·67
L2		15		2	1·00
L3		27		4	1·00
L4		1		0	1·00
Perianal involvement		16		3	0·67

Crohn's disease (CD) patients, healthy control (HC) volunteers and patients expressing low *OPTN* levels (OPTN^low^); gender, age at sampling ± SD, smoking status, current medication and phenotypes (L1 ileal, L2 colonic, L3 ileocolonic, L4 oral) are shown.

These studies were approved by the Joint UCL/UCLH Committees on the Ethics of Human Research (02/0324). Written informed consent was obtained from all volunteers. No patient was studied more than once in each of the different sets of experiments.

#### Monocyte-derived macrophage isolation

Peripheral venous blood samples were collected into heparin (5 U/ml). Mononuclear cells were isolated by differential centrifugation (900 ***g***, 30 min, 20°) over Lymphoprep® (Axis-Shield, Oslo, Norway) and washed twice with sterile PBS (GIBCO, Paisley, UK) at 300 ***g*** (5 min, 20°). Cells were resuspended in 10 ml RPMI-1640 (Invitrogen, Paisley, UK) supplemented with 100 U/ml of penicillin (GIBCO) and 100 μg/ml streptomycin (GIBCO) and 20 mm HEPES buffer (Sigma-Aldrich, Poole, UK) (RPMI), and plated at a density of approximately 5 × 10^6^ cells/ml in 8 cm^2^ Nunclon™ Surface tissue culture dishes (Nunc, Roskilde, Denmark). After an initial culture period of 2 hr at 37°, 5% CO_2_, the non-adherent cells were discarded and 10 ml of fresh RPMI supplemented with 10% fetal bovine serum (FBS) (Sigma-Aldrich) (10% FBS/RPMI) was added to each tissue culture dish. Cells were then cultured for 5 days at 37°, 5% CO_2_, with the addition of a further 10 ml fresh 10% FBS/RPMI after 24 hr. Adherent cells were scraped on day 5 and re-plated at 10^5^/well for cytokine analysis and 10^6^/well for RNA extraction in X-Vivo-15 medium (Cambrex, Walkersville, MD). These primary MDM were incubated overnight at 37°, 5% CO_2_ to adhere, and then stimulated for 4 hr (transcriptomic studies) or 24 hr (cytokine studies) with (100 : 1) heat-killed *Escherichia coli* clone NCTC 10418 (HkEc).

#### Cytokine secretion assay

Supernatant was collected from HkEc-stimulated MDM and an inflammatory cytokine assay was performed using a custom-designed human multiplex assay (TNF-*α*, IFN-*γ*, IL-6, IL-8 and IL-10) (Meso Scale Discovery, Rockvilla, MD) and read on a SECTOR® Imager 6000 (Meso Scale Discovery).

Release of TNF-*α* from THP-1 monocytic cells was determined using the L929 bioassay, as described previously.^9^ Interleukin-6 (R&D Systems, Abingdon, UK) and IL-10 (Peprotech, London, UK) release by THP-1 cells were determined using ELISAs.

Cytokine release in culture supernatants was normalized for the numbers of viable cells in each well, ascertained with the MTT (3-[4,5-dimethylthiazol-2-yl]-2,5-diphenyl tetrazolium bromide, tetrazolium salt) assay (Boehringer Ingelheim, Berkshire, UK). Twenty microlitres of 2·5 ng/ml MTT was added to each well and incubated for 4 hr at 37° in 5% CO_2_. Supernatants were carefully discarded and 100 μl/well of lysis solution (90% isopropanol, 0·5% SDS, 0·04 N HCl, and 10% H_2_O) was added to each well for 1 hr at room temperature. The absorbance was read at 570 nm using a microplate reader (FLUOstar OMEGA; BMG Labtech, Buckinghamshire, UK).

#### Whole genome microarray analysis

Total RNA was harvested from MDM using the RNeasy Mini Kit with RNase-free DNase treatment (Qiagen, Hilden, Germany), in accordance with the manufacturer's instructions. Optical density readings were determined for OD_260_/OD_280_ and OD_260_/OD_230_ using a NanoDrop ND-1000 spectrophotometer (Thermo Fisher Scientific, Hemel Hempstead, UK) to assess protein and solvent contamination, respectively. RNA integrity was analysed by assessing ribosomal RNA band 28S/18S ratios using high-resolution electrophoresis on an Agilent Bioanalyser (Agilent Technologies, Inc, Berkshire, UK) and attained an integrity score of > 8. Total RNA (500 ng) was amplified using the Illumina TotalPrep-96 RNA Amplification kit (Life Technologies Ltd, Paisley, UK), normalized to 150 ng/μl, and 750 ng was hybridized to Illumina Human-WG6 v3.0 Expression BeadChips (Illumina, Essex, UK) for 16 hr at 58°. Bead arrays were stained with streptavidin-Cy3 (GE Healthcare, Amersham, UK) and scanned using the Bead array reader and processed with genomestudio® data analysis software (Illumina). Data were smoothed with cubic spline normalization.

#### Microarray data analysis

Poor quality probes, as defined using the latest BioConductor^21^ Illumina annotation (illuminaHumanv4.db), were excluded. Only probes that were detected at *P* < 0·01 in at least 50% of the samples were retained. Batch effects were corrected using ComBat normalization with disease status as a covariate and efficacy of normalization was verified by principal component analysis.^22^ Differential gene expression analysis was conducted using limma^23^ with adjustment for multiple testing by the method of Benjamini and Hochberg.^24^

To detect subgroups of patients in whom expression profiles for individual genes were different from the control population, we used the z-score outlier detection (ZODET) method.^25^ This analysis aims to identify abnormally expressed genes (‘outliers’) in individuals and subgroups compared with a cohort of healthy controls (HC). Two adjustable thresholds were used to identify potential probe outliers: (i) the significance level (*P*-value) of the standardized deviation of the expression levels from the test sample when compared with the mean expression levels of the comparison group; this was set at *P* < 0·005; and (ii) the fold-change between the expression level from the test sample and the mean of the comparison group; this was set at 1 (on the log_2_ scale). All of the transcriptomic data used in this study have been deposited in the Gene Expression Omnibus (GEO) with accession no. GSE60083.

#### Statistical analysis of cytokine secretion data

Statistical significance of differences in cytokine secretion data was assessed using Student's *t*-test or paired *t*-test as appropriate. A *P*-value < 0·05 was considered significant.

Pairwise correlation analysis of all possible cytokine pairs was conducted with log_2_-transformed data separately for CD and HC. The extent of correlation was quantified by Pearson's correlation coefficient.

#### Real-time quantitative PCR analysis

RNA (1 μg) was converted to cDNA using oligo d(T) primers and reverse transcription using the Promega reverse transcription kit (Qiagen). Real-time quantitative PCR was performed using a SensiMix® No Ref DNA kit (Bioline reagents, London, UK), using peptidylprolyl isomerase A as endogenous control. All samples and standards were run in triplicate. Primers for optineurin (OPTN) were forward 5′-TGCCTGACATAGACACGTTAC-3′, reverse 5′-GGCAAAATATTTGAGTGAAACAA-3′, ADAMDEC1 forward 5′-CGTGTAAACTGAAGCCTGGA-3′, reverse 5′-TTCACAAGATTCCTGGGACAG-3′, commercially available primers for peptidylprolyl isomerase A and GAPDH were used as endogenous controls (Human Endogenous Control Gene Panel; Bioline). Relative expression was compared between groups using the ΔΔC_t_ method.

#### Sequencing of the OPTN genomic region

The reference sequence of the OPTN gene was obtained from the University of California Santa Cruz (UCSC) genome browser. Forward and reverse primers were designed for specific amplification of OPTN exons, promoter and downstream reactions (see Supporting information, Table S1). PCR was carried out using Hotstar Taq master mix (Qiagen GmbH) and 200 pmol of the appropriate forward and reverse primers (Eurofins MWG operon, Munich, Germany). PCR products were purified using the QIAquick PCR purification kit (Qiagen GmbH), in accordance with the manufacturer's instructions. Sequencing of the fragments was conducted at the Wolfson Institute for Biomedical Research, UCL by automated Sanger dideoxy sequencing reactions. Sequences were searched for previously recognized variants in the OPTN gene, as documented in the National Centre for Biotechnology Information (NCBI) SNP database.

#### Western blotting

Western blotting was used to determine intracellular OPTN levels. Cells were lysed in Laemmli sample buffer (0·06 m Tris–HCl pH 6·8, 2% SDS, 10% glycerol, 5% *β*_2_-mercaptoethanol (VWR, Leicestershire UK), 0·04% [weight/volume (w/v)] bromophenol blue, Complete protease inhibitor cocktail tablet (Roche Diagnostics GmbH, Mannheim, Germany), and phosphatase inhibitor cocktails 2 and 3 (Sigma-Aldrich). Proteins were separated by SDS–PAGE and transferred onto Hybond P nitrocellulose membrane (GE Healthcare, Buckinghamshire, UK) with a semi-dry transfer system (Trans-Blot SD semi-dry transfer cell; Bio-Rad, Hemel Hempstead, UK) in transfer buffer [200 mm glycine, 0·1% SDS (w/v), 10% methanol (v/v), 25 mm Tris–HCl pH 8·8]. Membranes were incubated overnight at 4° with primary antibodies directed against OPTN (HPA003360; Sigma-Aldrich, 1 : 1000) or *β*-actin (Sigma-Aldrich; 1 : 1000) and then with horseradish peroxidase-conjugated anti-rabbit IgG antibody (GE Healthcare, Amersham, UK, 1 : 2000) for 1 hr at room temperature, and developed using the Enhanced Chemiluminescence method (ECL PLUS; GE Healthcare). Densitometry analysis was performed using imagej software (National Institutes of Health, Bethesda, MA), with band densities normalized to *β*-actin.

#### Small interfering RNA knockdown in THP-1 cells

THP-1 cells (a human pro-monocytic cell line) were cultured in RPMI-1640 medium (Invitrogen), supplemented with 10% FBS (Sigma-Aldrich), 20 mm HEPES (Sigma-Aldrich), 100 U/ml penicillin, 100 μg/ml streptomycin (GIBCO) and 50 μm*β*_2_-mercaptoethanol (Invitrogen). OPTN expression in THP-1 cells was depleted by transfection of small interfering RNAs (siRNAs) targeted against OPTN, using the Amaxa Cell line Nucleofector® kit V (Amaxa/Lonza, Basel, Switzerland). THP-1 cells were harvested by centrifugation and washed with PBS. Cells were resuspended in Nucleofector® solution V, to obtain a concentration of 10^6^ cells/100 μl. For each transfection, 100 μl of cell suspension was mixed with 60 pmol of appropriate siRNAs. Silencer® pre-designed and validated siRNA directed against OPTN was used to deplete expression (ID # 4392420; Ambion, Austin, TX). Non-targeting Accell™ siRNA was also used as a negative control for off-target effects (catalogue no. D-001910- 01-05, Dharmacon; Thermo Fisher Scientific). Transfection was performed using an Amaxa Nucleofector® (programme U-01). Following transfection, cells were resuspended in RPMI-1640 medium (Invitrogen), supplemented with 10% FBS (Sigma-Aldrich), 20 mm HEPES (Sigma-Aldrich), 100 U/ml penicillin, 100 μg/ml streptomycin (GIBCO) and 50 μm*β*_2_-mercaptoethanol (Invitrogen) and cultured at 37° in an atmosphere of 5% CO_2_. Subsequent assays were performed 24 hr after transfection. For cytokine assays, 10^5^ cells were resuspended in 100 μl medium and stimulated for 6 hr with 2 μg/ml Pam_3_CSK_4_ (Alexis) or 20 HkEc/cell.

## Results

### Pro-inflammatory cytokine secretion from MDM is attenuated in CD

**Figure 1:**
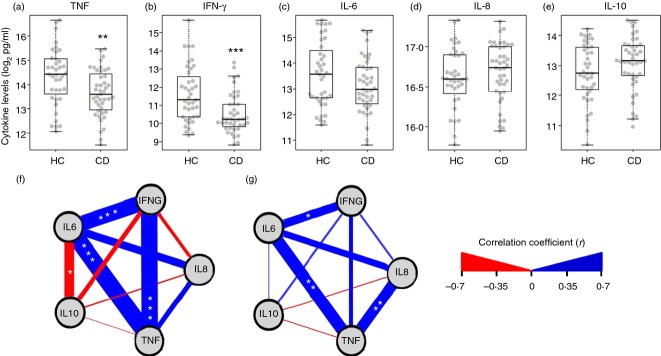
Inflammatory cytokine release from monocyte-derived macrophages (MDM) from patients with Crohn's disease (CD; *n* = 43) and healthy controls (HC; *n* = 39) after 24 hr heat-killed *Escherichia coli* clone NCTC 10418 (HkEc) stimulation. (a) Tumour necrosis factor (TNF), (b) interferon-*γ* (IFN-*γ*), (c) interleukin-6 (IL-6), (d) IL-8, and (e) IL-10. Graphical representation of the regression analysis performed using a Pearson's correlation on the cytokine secretion from MDM after 24 hr HkEc stimulation in both the (f) HC (*n* = 39) and (g) CD (*n* = 43) cohorts. Increased thickness of the interconnecting lines demonstrates the strength of the association (*r* value) and the colour denotes the direction (blue = positive and red = negative). *P < 0·05, **P < 0·01 and ***P < 0·001.

Given the important roles of TNF-*α*, IFN-*γ*, IL-6, IL-8 and IL-10 in acute inflammation and the previous association of these cytokines with CD, we determined their secretion by macrophages over a 24-hr period of stimulation with HkEc. The secretion of TNF-*α* (*P* = 0·0045) and IFN-*γ* (*P* = 0·0004) were significantly lower in the CD cohort (*n* = 43) compared with the HC (*n* = 39) (Fig. 1a,b) whereas equivalent amounts of IL-6, IL-8 and IL-10 were released in the two groups (Fig. 1c–e).

### Coordinated release of TNF-*α* and IFN-*γ* is disrupted in MDM from CD patients

Emerging evidence suggests that the post-translational processing and secretion of cytokines proceeds via a tightly regulated, complex process involving distinct, yet overlapping pathways.^17^ The precise molecules and processes involved are currently incompletely understood.^18^ To identify whether there is any evidence of co-ordinated cytokine secretion from MDM stimulated with HkEc, and whether abnormalities in this process could account for the defective release associated with CD, the correlation between all pairs of cytokines was examined in HC and CD separately. MDM from HC subjects demonstrated strong positive correlations between levels of secreted TNF-*α* and IFN-*γ* in response to HkEc stimulation (Fig. 1f) (*r* = 0·64, *P* = 9·5 × 10^−6^). There was also evidence of a negative correlation between IL-10 and IL-6 (*r* = −0·39, *P* = 0·01). Interleukin-8 was not significantly associated with any of the other cytokines tested. The strong association between TNF-*α* and IFN-*γ* was not evident in the MDM isolated from CD patients (Fig. 1g) (*r* = 0·15, *P* = 0·33). The IL-6 association with TNF-*α* (HC; *r* = 0·67, *P* = 2·8 × 10^−6^, CD; *r* = 0·47, *P* = 0·0016) and IFN-*γ* (HC; *r* = 0·60, *P* = 4·8 × 10^−5^, CD; *r* = 0·33, *P* = 0·03) was preserved in MDM from CD patients but the negative correlation with IL-10 (*r* = 0·02, *P* = 0·85) was lost. These results suggest a breakdown in the coordinated release of pro-inflammatory molecules in MDM derived from patients with CD after HkEc stimulation. Of note, the sample size in the CD group exceeded that of the HC group, indicating that the failure to detect correlations in CD was not due to insufficient power.

**Figure 2:**
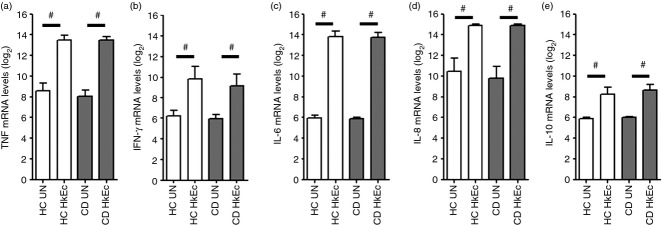
Cytokine gene induction after heat-killed *Escherichia coli* clone NCTC 10418 (HkEc) stimulation in the healthy controls (HC; *n* = 14) and Crohn's disease (CD; *n* = 30) subjects. (a) Tumour necrosis factor (TNF), (b) interferon-*γ* (IFN-*γ*), (c) interleukin-6 (IL-6), (d) IL-8, (e) IL-10 all demonstrated significant gene induction after HkEc stimulation. Cytokine mRNA levels were obtained from whole genome microarray data. There was no difference in the level of gene induction between the HC and CD cohorts. #*P* < 0·001.

The transcription of cytokine genes in genes of HC and CD MDMs was determined for both HC and CD before and after HkEc stimulation. In concordance with previous findings, the transcription of all these genes was increased by bacterial stimulation. No significant alteration in cytokine gene induction for TNF-*α*, IFN-*γ*, IL-6, IL-8 and IL-10 in MDM derived from CD patients compared with MDM from HC (Fig. 2), before or after HkEc stimulation, indicating that the defective secretion of TNF-*α* and IFN-*γ* has another cause.

### Outlier analysis of macrophage transcriptome identifies candidate genes responsible for abnormal cytokine secretion

**Figure 3:**
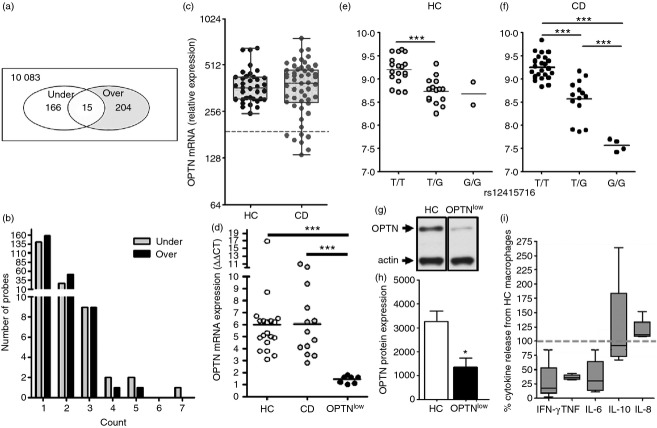
Gene expression analysis using microarray data from unstimulated monocyte-derived macrophage (MDM) from HC (*n* = 42, control group) and CD (*n* = 58, test group) subjects. (a) MDM were found to express 10 468 probes (detection *P*-value < 0·01 in > 50% of cases) of which < 3·5% were found to be expressed at abnormal levels (under- or over-expressed z-score *P* < 0·005 and a fold change of > 1) in any of the 58 CD patients tested. (b) The majority of the probes identified as abnormally expressed were unique to an individual. Only 11 and 14 probes were over- or under-expressed, respectively in three or more CD subjects tested. (c) The most commonly identified probe (ILMN_2381899), which corresponds to optineurin (OPTN), was under- expressed in seven patients out of the 58 tested. Each circle represents an individual's expression value for ILMN_2381899 overlaid with a box plot (mean plus 25th and 75th centile). Individuals that lie beneath the grey dotted line were classified as an outlier. (d) Quantitative PCR was used to verify the under-expression of OPTN in the individuals (OPTN^low^) identified as outliers (HC *n* = 19, OPTN^low^ *n* = 7 and CD *n* = 13). (e) OPTN gene expression in macrophages from HC by single nucleotide polymorphism (SNP) rs12415716 genotype. (f) OPTN gene expression in macrophages from CD by SNP rs12415716 genotype. (g) OPTN intracellular protein levels were determined using MDM whole cell lysates and Western blotting. (h) Quantification of the levels of intracellular OPTN in OPTN^low^ individuals (*n* = 4) compared with HC (*n* = 4). Individuals who demonstrated reduced OPTN gene expression were also found to have reduced OPTN protein levels compared with HC. (i) MDM cytokine secretion profile from OPTN^low^ patients (*n* = 4) stimulated with HkEc for 24 hr compared with the HC cohort (*n* = 43). OPTN^low^ individuals secreted reduced tumour necrosis factor (TNF), interferon-*γ* (IFN-*γ*) and interleukin-6 (IL-6) but equivalent levels of IL-8 and IL-10 in comparison to the HC population. Students *t*-test, and ***P < 0·001.

To interrogate potential molecular lesions that might be responsible for the impaired cytokine release in CD cells, microarray analysis was performed on unstimulated MDM. Direct comparisons of the transcriptome profiles of CD (*n* = 58) and HC (*n* = 42) macrophages did not reveal a difference between the two groups after correcting for multiple testing (Table 2 and see Supporting information, Table S2), which can be explained by the highly heterogeneous nature of the disease. In addition, the inability to separate macrophages from CD and HC subjects by principle component analysis provides evidence that the cellular phenotype arising from the culturing of the peripheral blood monocyte pool is similar in the two groups (data not shown). We hypothesized that subgroups of individuals within the CD population might share common abnormalities in gene expression which could influence cytokine secretion and searched for these using ZODET.^25^ This method enables the identification of gross abnormalities in gene expression in individual patients’ transcriptomes, which can be combined to identify potential subpopulations. MDM expressed genes that hybridized to 10 468 probes (detection *P*-value < 0·01 in > 50% of cases) of which only 385 (3·7%) were identified as expressed at significantly abnormal levels in one or more of the 58 CD patients tested when compared with the HC cohort (Fig. 3; see Supporting information Table S3). Of these 385 probes, 166 were found to be under-expressed and 204 over-expressed, with 15 probes being identified as both over- and under-expressed (Fig. 3a). The majority of probes identified were found in only one individual, but 25 probes were expressed at significantly abnormal levels in three or more CD subjects tested (Fig. 3b; Table 3). The most frequently identified probe, ILMN_2381899 corresponds to OPTN and was under-expressed in seven individuals with CD (12% of the CD patients tested) (Fig. 3c). OPTN has previously been associated with vesicle trafficking, autophagy induction and TNF receptor signalling.^26^–^30^ In addition to OPTN, ILMN_2103107 [ADAM-like, decysin 1 (ADAMDEC1)], ILMN_2160476 [chemokine (C-C Motif) Ligand 22 (CCL22)] and ILMN_1676256 (no annotated gene available) were identified as abnormally expressed in five of the 61 CD patients screened. Quantitative PCR confirmed the reduced expression of OPTN and ADAMDEC1 in the patients identified as under-expressing these genes using ZODET (Fig. 3d and data not shown).

**Table 2:** Differential gene analysis of microarray data comparing resting monocyte-derived macrophages (MDM) from healthy controls (HC; *n* = 42) and Crohn's disease patients (CD; *n* = 58)

Probe	Gene	HC mean	CD mean	Fold change	*P*-value	ADJP
ILMN_1694671	ZFAND2A	8·17	7·89	−0·28	9·83E-05	0·36
ILMN_1734184	PLBD2	8·43	8·61	0·18	0·0001458	0·36
ILMN_1808356	FAM3A	9·12	9·31	0·20	0·0002436	0·36
ILMN_1706498	DSE	9·00	8·81	−0·19	0·000373	0·36
ILMN_1811373	FAM20B	7·99	8·09	0·10	0·0004633	0·36
ILMN_1672662	SLC20A1	10·35	10·15	−0·19	0·0004851	0·36
ILMN_1671054	HLA-A	11·74	11·41	−0·33	0·0005167	0·36
ILMN_1730201	DTNA	7·17	7·40	0·23	0·0005298	0·36
ILMN_1773485	QKI	7·33	7·47	0·14	0·0005452	0·36
ILMN_1755134	HSPA4	6·59	6·68	0·09	0·0005542	0·36
ILMN_1741727	QPCT	10·37	9·85	−0·52	0·000598	0·36
ILMN_1803483	KIAA2013	8·86	9·00	0·15	0·0006012	0·36
ILMN_1807981	SIGIRR	6·33	6·44	0·11	0·0006271	0·36
ILMN_1714433	MARCKSL1	7·28	6·95	−0·33	0·0006424	0·36
ILMN_1796669	PSEN1	6·28	6·37	0·09	0·0006703	0·36
ILMN_1726967	TWSG1	6·93	7·02	0·08	0·0006712	0·36
ILMN_2160476	CCL22	9·38	8·72	−0·66	0·0006893	0·36
ILMN_1651819	GALNT11	8·08	8·21	0·13	0·0007314	0·36
ILMN_2400500	CERS2	7·77	7·91	0·13	0·0008091	0·36
ILMN_2212763	ICAM3	7·24	6·95	−0·29	0·0008527	0·36

There were no significant alterations in gene expression between HC and CD after correcting for multiple testing (ADJP, significance *P* < 0·05). The top 20 probes ranked by uncorrected *P*-values are illustrated along with the mean expression levels in both HC and CD cohorts, fold change between the HC and CD populations and the *P*-value after correction for multiple testing (ADJP). The complete analysis can be found in the Supporting information, Table S2.

**Table 3:** Gene outlier analysis using microarray data from unstimulated monocyte-derived macrophages (MDM) from healthy controls (HC; *n* = 42) and Crohn's disease patients (CD; *n* = 58)

Probe	Gene	Under-expressed	Over-expressed
ILMN_2381899	OPTN*	7	0
ILMN_2103107	ADAMDEC1*	5	0
ILMN_2160476	CCL22	5	0
ILMN_1676256	TPSAB1	0	5
ILMN_1703538	AIF1	4	0
ILMN_1716909	ADAMDEC1	4	0
ILMN_1687757	AKR1C3	0	4
ILMN_2092118	FPR1	3	2
ILMN_1671568	ECHDC2	3	0
ILMN_1749403	TSPAN33	3	0
ILMN_1765332	TIMM10	3	0
ILMN_1782729	CLECL1	3	0
ILMN_1797875	ALOX5AP	3	0
ILMN_1815895	LOC649143	3	0
ILMN_2262288	EEF1G	3	0
ILMN_2341815	TFG	3	0
ILMN_1668039	GYPC	0	3
ILMN_1668134	GSTM1	0	3
ILMN_1670490	PDPN	0	3
ILMN_1710124	CMTM8	0	3
ILMN_1715401	MT1G	0	3
ILMN_1716218	RPS6KA2	0	3
ILMN_1752965	GREM1	0	3
ILMN_1810420	DYSF	0	3
ILMN_2169801	TPSAB1	0	3

All of the probes that were identified as reaching outlier significance (under- or over-expressed z-score *P* < 0·005 and a fold change of > 1) in three or more CD subjects are shown. A full list of all outliers identified in this analysis can be found in the Supporting information; Table S3.

*altered gene expression confirmed by qPCR.

### Reduced OPTN expression associated with a specific haplotype

Patients with CD who were identified as expressing low OPTN levels (OPTN^low^) were subjected to DNA sequencing across all 16 exons and approximately 2K bases up- and downstream of the OPTN gene. No novel mutations were detected in these individuals and all sequence variations identified had been previously documented in the 1000 Genomes Project (data not shown).^31^ Five of the seven OPTN^low^ subjects were found to be homozygous for the single nucleotide polymorphism (SNP) rs12415716 (G/G, minor allele frequency 0·183) (see Supporting information, Fig. S1). The two other OPTN^low^ patients were heterozygous (T/G) for this SNP. The genotype of the remaining CD and HC cohort was determined and plotted against OPTN expression (Fig. 3e,f). The rs12415716 SNP was found to be an expression quantitative trait locus (eQTL) for OPTN. The presence of rs12515716 minor allele G in the CD cohort (coefficent = −0·78, *r*^2^ = 0·74 and *P*-value = 2·0 × 10^−13^) produced over twice the effect on OPTN gene expression compared with the HC population (coefficient = −0·37, *r*^2^ = 0·37 and *P*-value = 9·0 × 10^−5^). This difference is driven predominately by the presence of the OPTN^low^ outliers in the CD group. Using the 1000 Genomes database and a linkage disequilibrium threshold of 0·8 (*r*^2^) revealed that the SNP rs12515716 tags a region on chromosome 10 spanning 13 200 041–13 222 655, which contains the OPTN exons 7–16 (Fig. S1b–d). These results demonstrate that the low OPTN level identified in the seven CD subjects is partially due to the inheritance of a haplotype tagged by the rs12415716 SNP.

### Patients with low OPTN expression have reduced intracellular protein levels and release diminished amounts of TNF-*α*, IFN-*γ* and IL-6 after HkEc stimulation

The reduced expression of OPTN in seven CD patients identified by microarray analysis was verified by quantitative PCR (Fig. 3d). MDM were re-cultured from four of these patients 6–12 months after original sampling and they were found to consistently under-express OPTN (data not shown). In addition to having low transcription levels, the OPTN^low^ subjects were shown to have reduced intracellular OPTN protein levels compared with HC (Fig. 3g,h) and secreted reduced levels of TNF-*α*, IFN-*γ* and IL-6 compared with the HC cohort after HkEc stimulation (Fig. 3i). In contrast, secreted levels of IL-10 and IL-8 by MDM from CD and HC were similar (Fig. 3i). In addition to OPTN^low^ CD subjects the cytokine secretion profile of four of five individuals identified as low for ADAMDEC1 were also tested (see Supporting information, Fig. S2a). These subjects released reduced IFN-*γ* and TNF-*α* but similar levels of IL-6, IL-8 and IL-10 to the HC cohort. Further work will be required to determine the role ADAMDEC1 plays in macrophage biology.

### Specific knock-down of OPTN results in diminished TNF-*α* secretion after HkEc and Pam_3_CSK_4_ exposure

**Figure 4:**
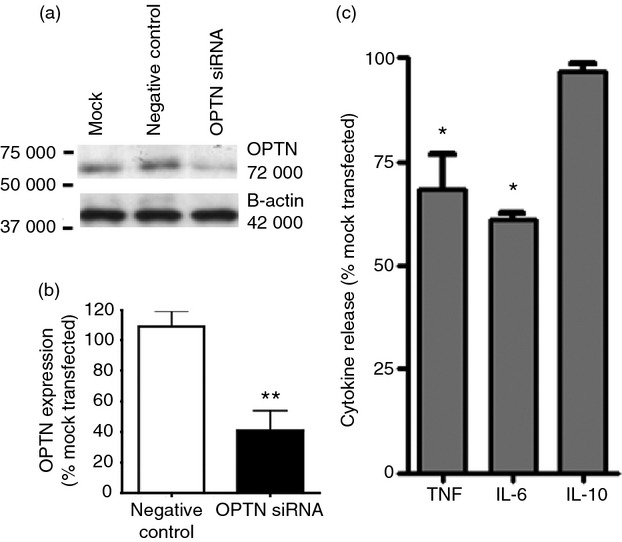
Knockdown of optineurin (OPTN) by small interfering RNA (siRNA) results in reduced cytokine secretion after bacterial stimulation. (a) Western blot analysis of OPTN levels in THP-1 cells 24 hr after siRNA treatment. (b) Quantification of the OPTN protein level 24 hr after siRNA treatment (*n* = 6). (c) Changes in cytokine secretion resulting from reduced OPTN expression after heat-killed *Escherichia coli* clone NCTC 10418 (HkEc) exposure was determined. ELISAs were used to calculate the effects on tumour necrosis factor (TNF), interleukin-6 (IL6) and IL-10 secretion after OPTN knockdown. Paired students *t*-test *P < 0·05 and **P < 0·01.

THP-1 mononuclear cells were transfected with siRNA against OPTN and stimulated with HkEc. OPTN protein levels were reduced by approximately 60% 24 hr after siRNA transfection (Fig. 4a,b). OPTN knockdown did not alter THP-1 viability any more than treatment with mock and negative siRNA (data not shown). OPTN-deficient THP-1 cells released significantly lower levels of TNF-*α* and IL-6 but normal levels of IL-10 after HkEc stimulation compared with mock-transfected cells (Fig. 4c). OPTN-deficient THP-1 cells were also shown to secrete reduced levels of TNF-*α* downstream of TLR2 activation (Fig. S2b). These results suggest that reduced OPTN levels have a direct effect on cytokine secretion and could contribute to the diminished levels identified in a subgroup of CD patients.

## Discussion

The evidence to support a link between abnormalities in the innate immune response and CD is rapidly expanding.^6^ Defective bacterial recognition through mutations in *NOD2* and anti-bacterial responses such as autophagy activation have been shown to be abnormal in CD.^6^–^9^,^13^

In this study we provide further evidence of defective secretion of pro-inflammatory cytokines by macrophages from patients with CD. We have demonstrated a strong association between secretion of TNF-*α*, IFN-*γ* and IL-6 proteins from healthy control MDM in response to *E. coli*, which is disrupted in CD. These results, combined with previous findings, suggest that alterations in the secretory pathways in the macrophages are responsible for the diminished cytokine release and subsequent defective acute inflammatory response observed in individuals with CD.^7^–^9^,^32^

Cytokines and chemokines released from macrophages play a key role in the regulation of the immune response, acting as messengers that regulate the initialization and finally the resolution of the inflammatory response to pathogens and acute injury. TLR and NLR signalling pathways, which result in cytokine gene activation, have been extensively studied,^10^,^11^ but little information is currently available on the mechanisms that control the post-translational processing and release of cytokines. To add further complexity, evidence suggests that secretory processes may differ between cell types. In this study we have used MDM and identified an association between TNF-*α*, IFN-*γ* and IL-6 at the level of secretion after *E. coli* stimulation. A number of studies have recently described an overlap in the secretory pathways for these three cytokines.^17^,^18^,^33^,^34^ Both TNF-*α* and IFN-*γ* were shown to share a secretory pathway involving the recycling endosome-associated molecules vesicle associated membrane protein 3 (VAMP3) and Rab11a in natural killer cells. VAMP3 was also co-localized with both TNF-*α* and IL-6 in the recycling endosome in activated RAW264.7 a mouse macrophages cell line. A single nucleotide polymorphism (rs35675666) that tags a chromosomal region that includes VAMP3 has been identified in patients with inflammatory bowel disease.^4^ An alteration in the secretory process involving VAMP3 may be partially responsible for the cytokine observations in this study.

Interestingly, the secreted levels of IL-10 and IL-8 appear unrelated to the other cytokines measured in this study, which may be due to the presence of alternative secretory mechanisms. These alternative pathways seem to be relatively unaffected in MDM from patients with CD. The molecular machinery involved in these alternative pathways is still unclear. Recently, IL-10 was shown to possess two distinct exit strategies, one via the recycling endosomes and a second post-Golgi route that also transports apolipoprotein E.^35^ Pathways associated with IL-8 release from macrophages remain less well defined. One study using HL60 human promyelocytic leukaemia cells reported a secretory pathway for IL-8 that involved an autophagy-related structure termed the TOR-autophagy spatial coupling compartment (TASCC).^36^ It is unclear if the TASCC structure is present in the MDM used in our study or if this is the only secretory pathway used by IL-8. Further studies are needed to unravel the complexities associated with macrophage cytokine secretion.

To identify the molecular basis of defective cytokine trafficking we have applied an outlier gene analysis strategy, which has previously been successfully used in identifying heterogeneous molecular aberrations in certain cancers.^37^ This approach yielded a relatively small number of genes that were expressed at significantly altered levels in the MDM from subgroups of CD patients. The most frequently under-expressed gene was the multifunctional adapter protein OPTN. Functional studies on OPTN revealed a potential role in cytokine secretion from macrophages after bacterial and TLR activation. OPTN has been previously implicated in vesicular trafficking from the Golgi to the plasma membrane.^28^ The precise role attributed to OPTN seems to be cell-type-specific and studies on its role in human macrophages have not been reported. One study using mouse bone-marrow-derived macrophages identified a role for OPTN in TANK-binding kinase 1 (TBK1) activation and IFN-*β* production.^26^ A recent study has demonstrated that mutations in the ubiquitin binding site of OPTN (Optn^470T^) in mice results in the alteration of IFN-*β* gene induction through reduced IFN response factor 3 activation, but did not affect the secretion of TNF-*α* after lipopolysaccharide stimulation.^27^ These findings suggest that either the ubiquitin binding site in OPTN is not required for TNF-*α* secretion or there is a fundamental difference between mouse and human macrophages. The other potential explanation is a difference in stimuli used between our studies and the ones reported. The use of whole bacteria and the TLR2 ligand Pam_3_CSK_4_ may result in a different requirement for OPTN in the downstream processes.

Heterozygous mutations in OPTN have previously been linked to glaucoma and amyotrophic lateral sclerosis, although sequencing all exons of the OPTN gene in all seven patients identified as outliers revealed no such mutations, and no patients had any clinical features of glaucoma or neurodegenerative disease (data not shown).^38^ We were able to identify a haplotype that demonstrated a strong correlation with OPTN expression. The haplotype was tagged by the SNP rs12415716 which has a reported minor allele frequency of 0·18. rs1751262, which is in perfect linkage disequilibrium with rs12415716, has been shown to effect OPTN expression in fibroblasts.^39^ The effect on OPTN expression in individuals who are homozygous for the minor haplotype seems to be more pronounced in the CD patients than in HC. The reason for the difference in effect between individuals is still unclear, but could involve epistasis, whereby distant polymorphisms directly influence the effects of other polymorphisms on gene expression.^40^ A recent study by Hemani *et al*. described this phenomenon in peripheral blood cells and identified 129 genes that were influenced by three to 14 SNPs located on multiple chromosomes. This study highlights the complexity of gene expression and the potential impact that epistasis could have on human traits and diseases. We note that there is no association between this eQTL SNP and CD risk, as assessed by genome-wide association studies. Further studies will be required to ascertain the precise role played by OPTN in macrophage biology and cytokine secretion, and the reason for defective OPTN expression in these patients. To confirm an association between the under-expression of OPTN (and the additional genes reported) and susceptibility to CD, further replication cohorts will be required.

The studies presented here build on the mounting evidence of a defective innate immune response in individuals who develop CD. Defective cytokine secretion identified in patients with CD is restricted to a subset of cytokines that seem to share overlapping intracellular trafficking pathways. Alterations in the expression levels or mutations in the proteins involved in the complex trafficking pathways could result in the reduced cytokine secretion observed. The inability to release sufficient levels of potent pro-inflammatory mediators will potentially alter the ensuing inflammatory response. The implementation of a gene outlier analysis has identified a number of potential genes that could have a role in cytokine trafficking and processing in macrophages.

## Supporting Information

**Figure S1.** Haplotype associated with the reduced optineurin (OPTN) mRNA expression identified in patients with Crohn's disease.

**Figure S2.** Monocyte-derived macrophage (MDM) cytokine secretion profile from ADAMDEC1^low^ patients (*n* = 4) stimulated with heat-killed *Escherichia coli* clone NCTC 10418 (HkEc) for 24 hr compared to the healthy control (HC) cohort (*n* = 39).

**Table S1.** List of primers used to sequence the optineurin (OPTN) gene region.

**Table S2.** Differential gene analysis comparing resting monocyte-derived macrophages (MDM) from healthy controls (HC; *n* = 42) and patients with Crohns's disease (CD (*n* = 58).

**Table S3.** List of probes identified as under- or over-expressed in one or more Crohn's disease (CD) patient (*n* = 61) compared with the healthy control (HC) cohort (*n* = 43).
