# Supplementary figures and images for: Disruption of macrophage pro-inflammatory cytokine release in Crohn's disease is associated with reduced optineurin expression in a subset of patients
Source: Immunology. 2014 Dec 8;144(1):45–55. doi: 10.1111/imm.12338 (PMC4264909; doi:10.1111/imm.12338)

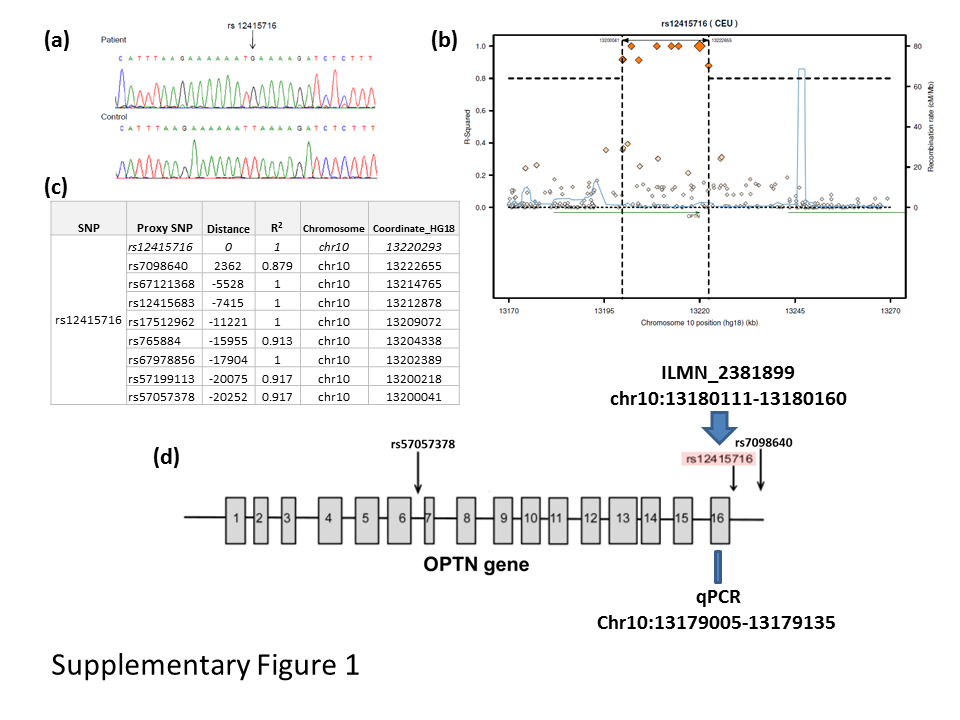

Supplement: Supplementary file 1 — Figure S1. Haplotype associated with the reduced optineurin (OPTN) mRNA expression identified in patients with Crohn's disease. [file imm0144-0045-sd1.tif]

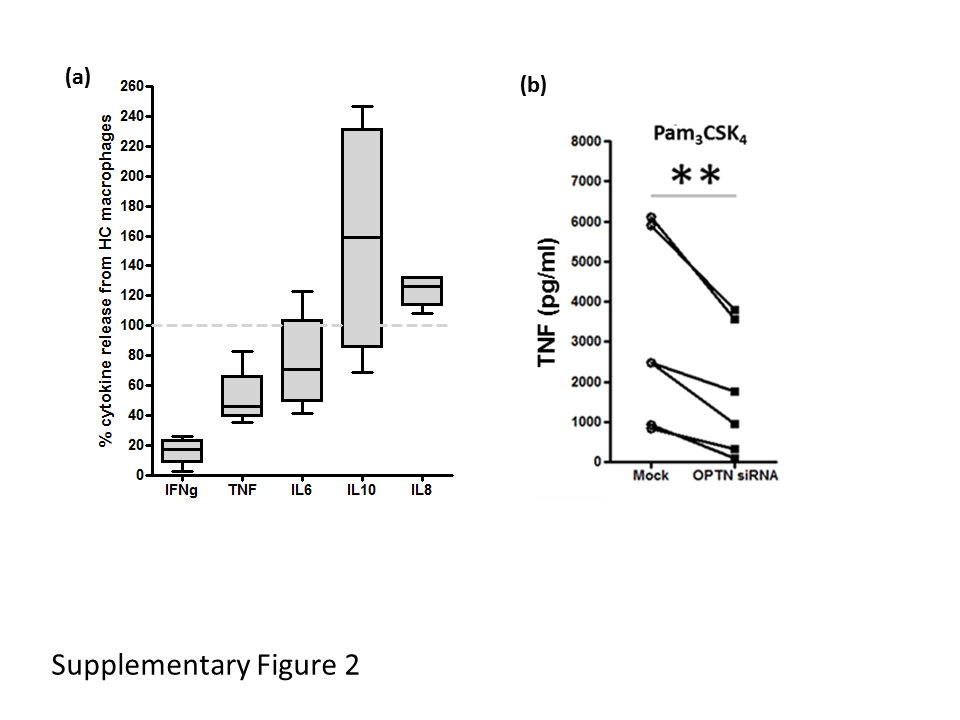

Supplement: Supplementary file 2 — Figure S2. Monocyte-derived macrophage (MDM) cytokine secretion profile from ADAMDEC1low patients (n = 4) stimulated with heat-killed Escherichia coli clone NCTC 10418 (HkEc) for 24 hr compared to the healthy control (HC) cohort (n = 39). [file imm0144-0045-sd2.tif]

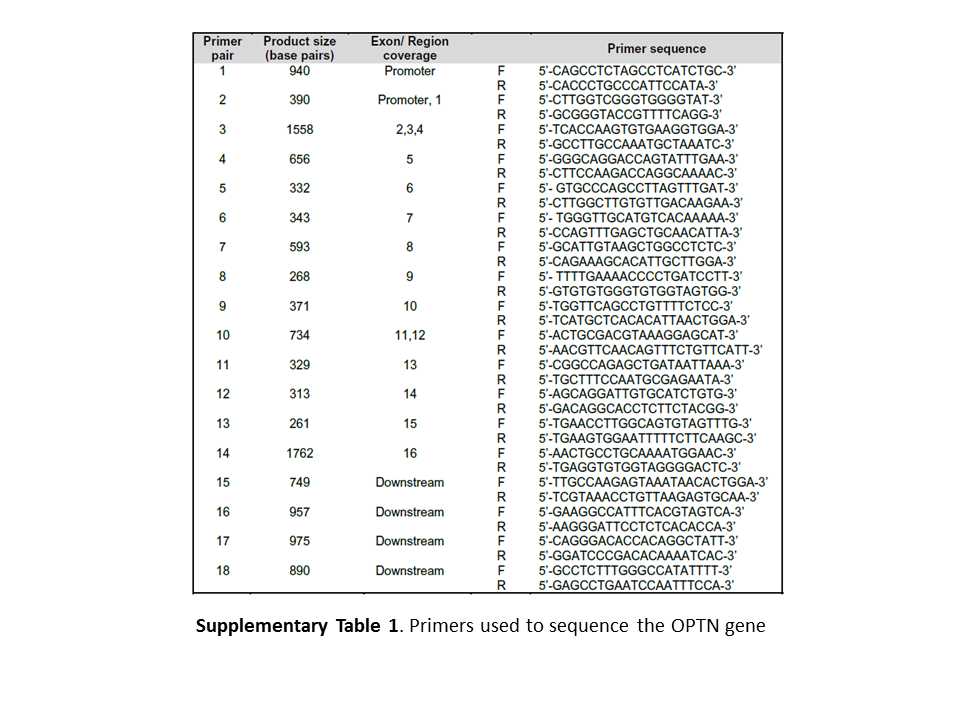

Supplement: Supplementary file 3 — Table S1. List of primers used to sequence the optineurin (OPTN) gene region. [file imm0144-0045-sd3.tif]
